# Clear identification of the rare solitary external iliac lymph node metastasis of testicular cancer by using indocyanine green fluorescence guidance

**DOI:** 10.1002/iju5.12273

**Published:** 2021-02-24

**Authors:** Yuki Enei, Fumihiko Urabe, Jun Miki, Kosuke Iwatani, Akira Hisakane, Keiji Yasue, Takafumi Yanagisawa, Takahiro Kimura, Shin Egawa

**Affiliations:** ^1^ Department of Urology The Jikei University Kashiwa Hospital Kashiwa Chiba Japan; ^2^ Department of Urology The Jikei University School of Medicine Tokyo Japan

**Keywords:** indocyanine green fluorescence, lymphatic drainage route, lymphatic metastasis, testicular cancer

## Abstract

**Introduction:**

There are few reports on indocyanine green fluorescence‐guided surgery in testis‐related diseases.

**Case presentation:**

A 38‐year‐old man underwent orchiectomy for left testicular cancer. Pathological diagnosis was pT1 seminoma. Seven years after the surgery, solitary left external iliac lymph node metastasis was suspected. We decided to perform laparoscopic lymph node dissection combined with indocyanine green fluorescence injection. During the operation, we injected indocyanine green fluorescence into his left inner inguinal ring and found that the lymph node was directly drained from the injection point. The pathological diagnosis of the indocyanine green fluorescence‐positive left external iliac lymph node was testicular cancer metastasis.

**Conclusion:**

We experienced a case of solitary left external iliac lymph node recurrence in testicular cancer. Using indocyanine green fluorescence injection, we could visualize the lymphatic drainage route, which helped us identify the lymph node as the primary landing site of metastasis.

**Abbreviations & Acronyms**
ICG: indocyanine green fluorescence
LN: lymph node

**Keynote message**

Solitary external iliac LN recurrence in testicular cancer is a rare form of metastasis. Using ICG injection during LN dissection, we could visualize the lymphatic drainage route, which helped us identify the primary landing site of metastasis.

## Introduction

Lateral and preaortic LNs are defined as the primary landing site of testicular cancer metastasis.^1^ To date, several reports have shown atypical lymphatic metastasis in testicular cancer.^2^, ^3^, ^4^ However, their main metastatic sites were inguinal LNs, and solitary external iliac LN metastasis is a rare pattern.^2^, ^5^

ICG is a fluorescent molecule that emits near‐infrared light. Recent reports of ICG imaging‐guided techniques have described the feasibility and safety of the detection of lymphatic drainage routes in various types of cancers.^6^, ^7^, ^8^

Here, we report a case of solitary left external iliac LN recurrence of testicular cancer. Using ICG injection, we identified the drainage route and dissected the LN as the primary landing site of LN metastasis.

## Case presentation

**Fig. 1:**
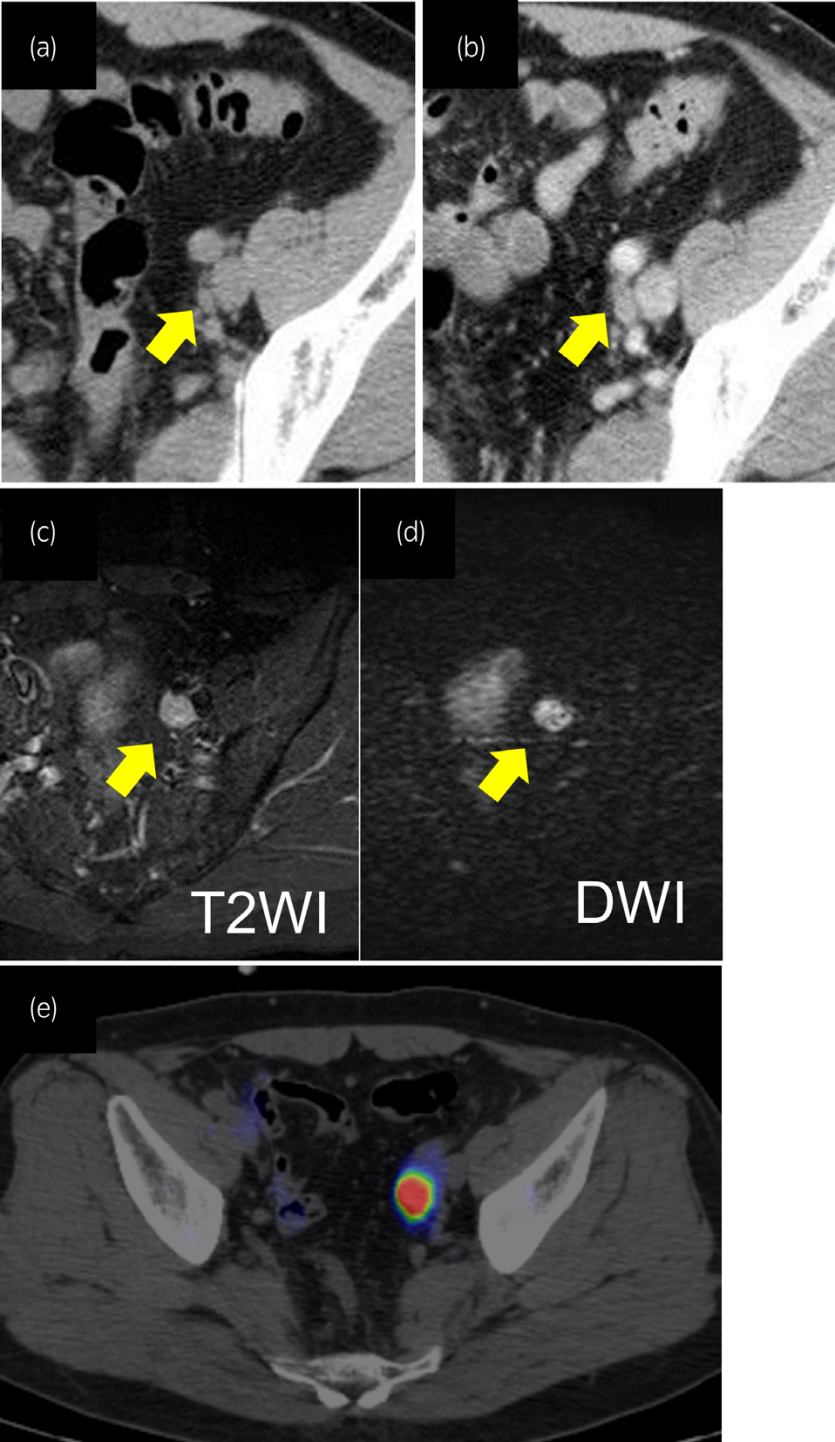
Imaging findings of left external LN swelling in a patient with testicular seminoma after left orchiectomy. LN swelling at (a) 7 years and (b) 9 years after orchiectomy. The lesion had a (c) high T2 image signal intensity and (d) high diffusion‐weighted imaging signal intensity. (e) Positron emission tomography/computed tomography fusion imaging revealed the accumulation of 18F‐labeled fluorodeoxyglucose in the left external LN.

A 38‐year‐old man with a history of right hernioplasty underwent left orchiectomy for testicular cancer. The pathological diagnosis was pure seminoma (pT1N0M0, stage 1). Serum testicular tumor markers, including lactate dehydrogenase, human chorionic gonadotropin and alpha‐fetoprotein, at diagnosis were all negative. Seven years after the surgery, left external iliac LN swelling was noted (Fig. 1a). All testicular tumor markers were negative, and no primary tumor was detected by clinical manifestation or imaging examination. Therefore, careful follow‐up was continued. However, the LN showed enlargement in size during the 4‐year follow‐up (Fig. 1b); thus, magnetic resonance imaging was performed. The lesion had a high T2‐weighted signal intensity and high diffusion‐weighted imaging signal intensity (Fig. 1c,d). Additionally, although testicular tumor markers were still negative, positron emission tomography scans depicted intense ^18^F‐fluorodeocyglucose uptake in the lesion (Fig. 1e). From these results, LN metastasis of testicular cancer was suspected.

**Fig. 2:**
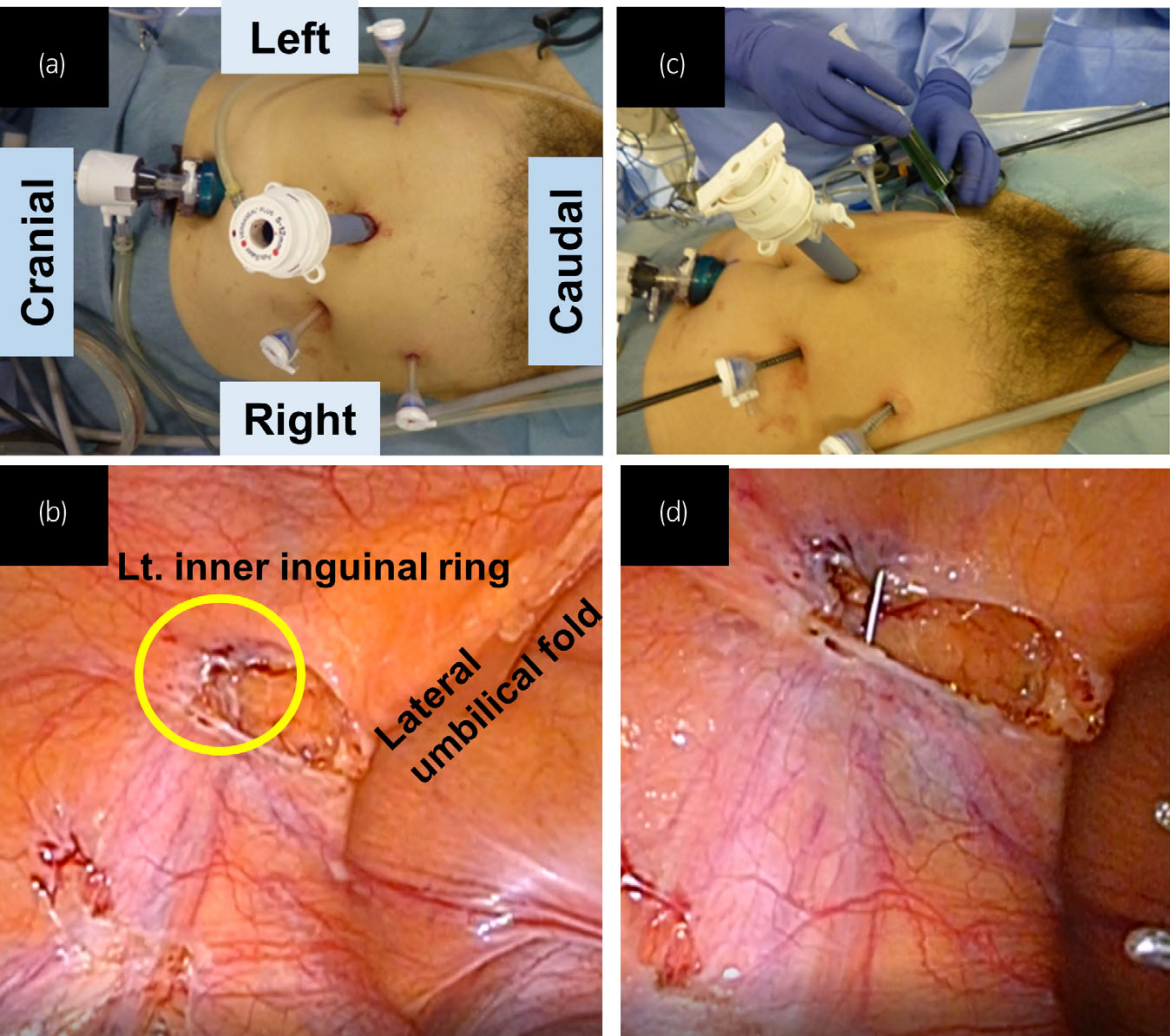
(a) Port arrangement for laparoscopic LN dissection. (b) The left inner inguinal ring is circulated in yellow. (c) ICG was percutaneously injected into the left inner inguinal ring. (d) Laparoscopic view of the injection.

**Fig. 3:**
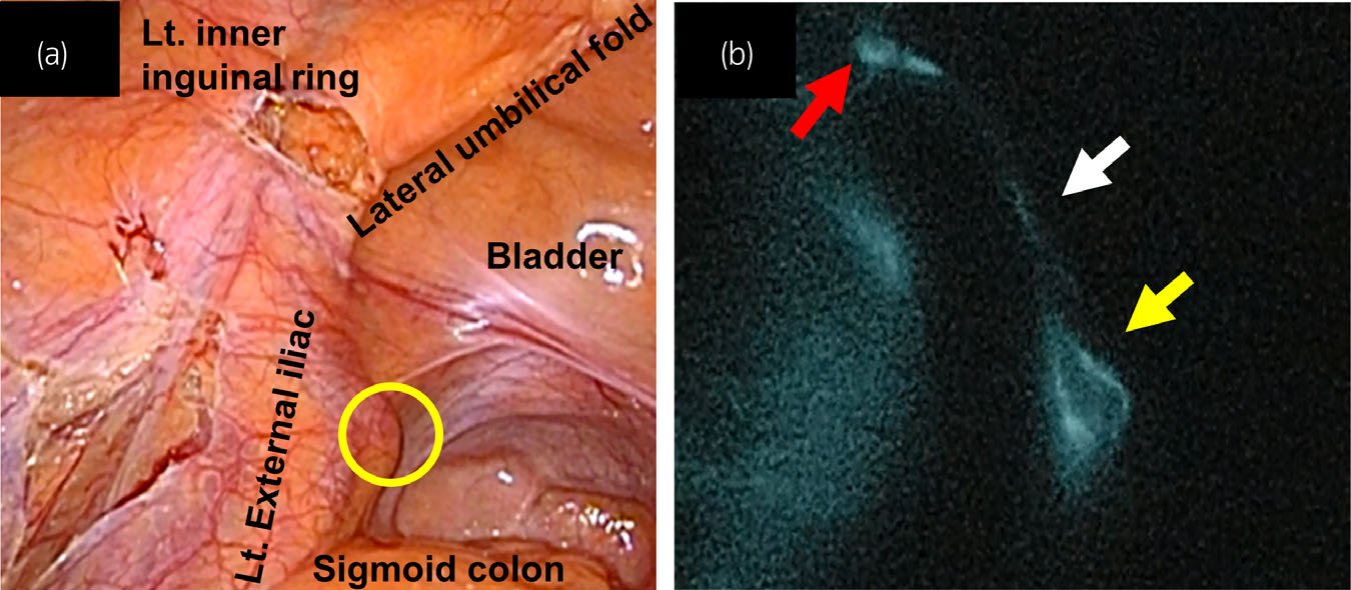
(a) Laparoscopic view of the left external iliac LN with white light, and the LN is circled in yellow. (b) The LN is directly drained from the left inner inguinal ring under ICG fluorescence endoscopy after injection of ICG. The red arrow shows the inguinal ring, the yellow arrow shows the external iliac LN, and the white arrow shows the lymphatic drainage route.

**Fig. 4:**
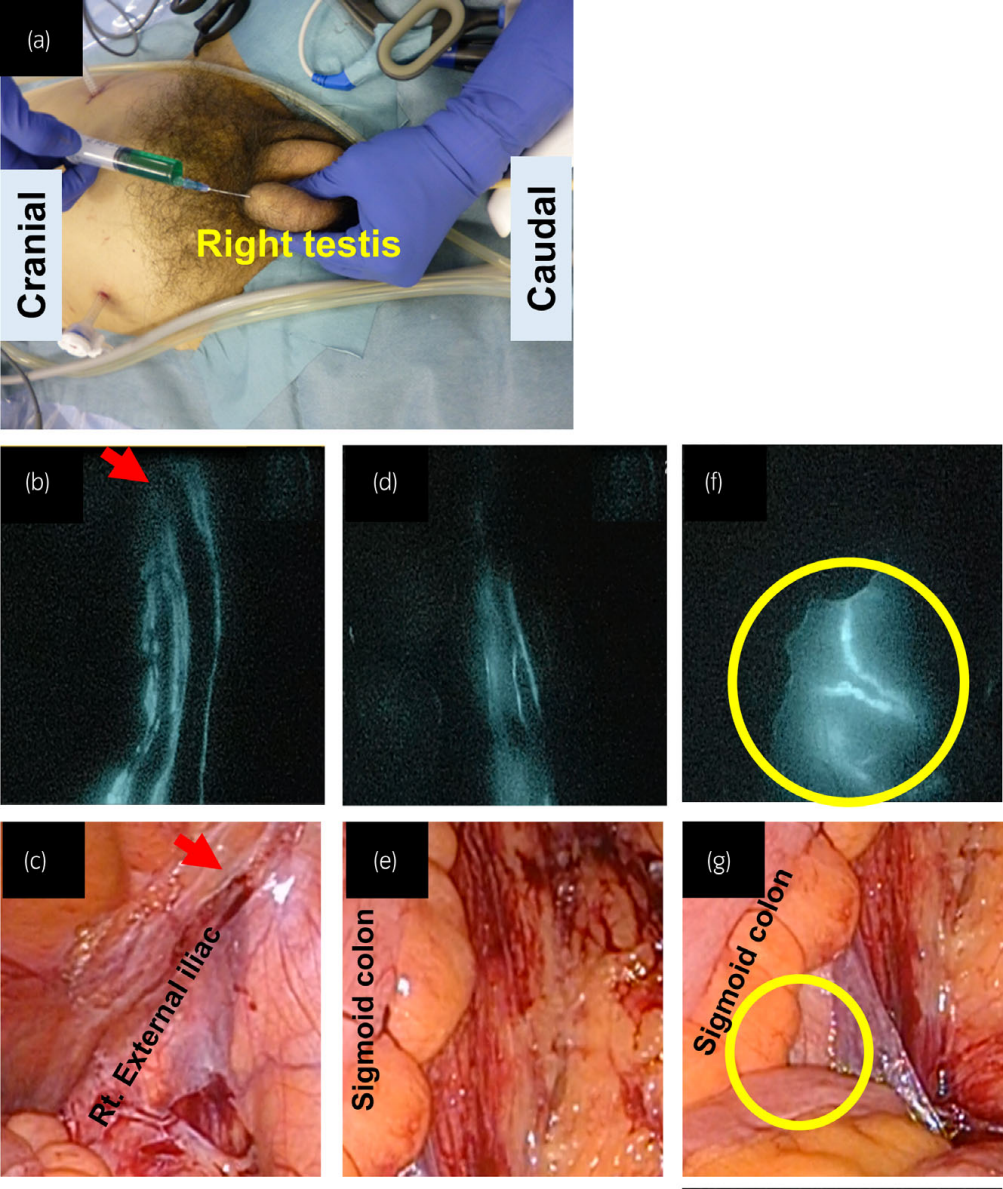
(a) ICG was injected into the right testis. (b,c,d,e) No abnormal lymphatic drainage route is shown on the right side under white light or ICG fluorescence endoscopy. The red arrow shows the right inner inguinal ring. (f,g) The para‐aortic LN was detected.

As solitary external iliac LN recurrence in testicular cancer is a rare form of metastasis, we decided to perform laparoscopic LN dissection combined with ICG injection. The patient was placed at a 15° Trendelenburg position, and a five‐port (5–10 mm) transperitoneal approach was employed (Fig. 2a). We percutaneously injected 1.5 mL of 0.25 mg/mL ICG into his left inner inguinal ring as the lymphatics exit testis through the inguinal ring to the lateral and preaortic LN (Fig. 2b–d).^9^ Then, we observed the lymphatic drainage route under a laparoscopic near infrared fluorescence imaging system. The left external iliac LN was directly drained from the injection point (Fig. 3a,b). We resected the swelling and lighting LNs, and additional left pelvic LN (left common iliac LNs and ICG negative left external iliac LNs) dissection was performed. Additionally, to examine the lymphatic drainage route from the right testis, we injected 3 mL of 0.25 mg/mL ICG into the right testis (Fig. 4a). No abnormal lymphatic drainage routes were noted on the right side (Fig. 4b–e).

Pathological diagnosis of the ICG‐positive left external iliac LN was metastasis of testicular cancer (1/1). No metastasis was detected in the other pelvic LNs, including left common iliac LNs (0/8) and ICG‐negative left external iliac LNs (0/2). There was no evidence of local recurrence or distant metastasis during the 6‐year follow‐up without any additional treatment.

This study was approved by the Ethics Committee of The Jikei University School of Medicine, Tokyo, Japan (25‐152‐7287). Written informed consent was obtained from the patient for publication of this article.

## Discussion

The interesting point of this case report is that we could directly observe the lymphatic drainage route and detect the LN using ICG injection. To date, several reports have shown the feasibility of the sentinel LN approach in testicular cancer using 99mTc‐nanocolloid.^10^, ^11^, ^12^, ^13^ Compared to 99mTc‐nanocolloid, the strong point of ICG images is not only depicting the LNs but also visualizing the drainage route in real time. In the present study, only the external iliac LN swelled, and it was visualized to be drained from the inner inguinal ring. Thus, we dissected the LN as the primary landing site of the LN, and it was the only metastatic LN.

To date, several reports have shown unusual lymphatic spreading of testicular cancer. In these cases, orchidopexy, varicocelectomy, or hernioplasty creates unusual lymphatic drainage routes, which mainly cause inguinal LN metastasis.^2^, ^14^ These metastases can be explained by the alteration of the normal lymphatic drainage route during the surgical approach and tissue healing after surgery.

In the present case, the patient had a history of right hernioplasty but not left hernioplasty. There are two potential explanations for this metastasis. The first is the disruption of lymphatic drainage by tumor infiltration. The second is that the patient constitutionally had an unusual lymphatic drainage route from the left testis to the external iliac LN. However, the pathological diagnosis of the primary tumor was not invasive, and we could not obtain a definitive explanation for this abnormal lymphatic drainage pattern.

The induction of chemotherapy is by far the most utilized salvage therapy for relapse in clinical stage 1 seminoma patients.^15^ As for additional treatment, although adjuvant chemotherapy was considered, follow‐up was decided after discussion with the patient. With careful observation, there was no recurrence 6 years after LN dissection. Further reports are needed, this case may provide us with the potential of dissecting LNs to control solitary LN recurrence in testicular cancer.

Additionally, we examined the right side of the lymphatic drainage route by ICG injection into the right testis. This is the first report to visualize the direct lymphatic drainage route from the testis to the para‐aortic LN, which is regarded as the first landing site of metastatic disease. This result showed the potential of ICG imaging in the detection of sentinel LNs in testicular cancer.

In conclusion, we experienced a case of solitary left external iliac LN metastasis in testicular cancer. Using ICG injection during LN dissection, we observed that the LN was directly drained from the inner inguinal ring, which helped us dissect the LN as the primary landing site of LN metastasis.

## Conflict of interest

The authors declare no conflict of interest.
